# Supplementary material for: The P-Site Loop of the Universally Conserved Bacterial Ribosomal Protein L5 Is Required for Maintaining Both Translation Rate and Fidelity
Source: Int J Mol Sci. 2023 Sep 19;24(18):14285. doi: 10.3390/ijms241814285 (PMC10531944; doi:10.3390/ijms241814285)
Supplement: Supplementary file 1 [file ijms-24-14285-s001.zip › Supplementary figures.pptx]

## Slide 1
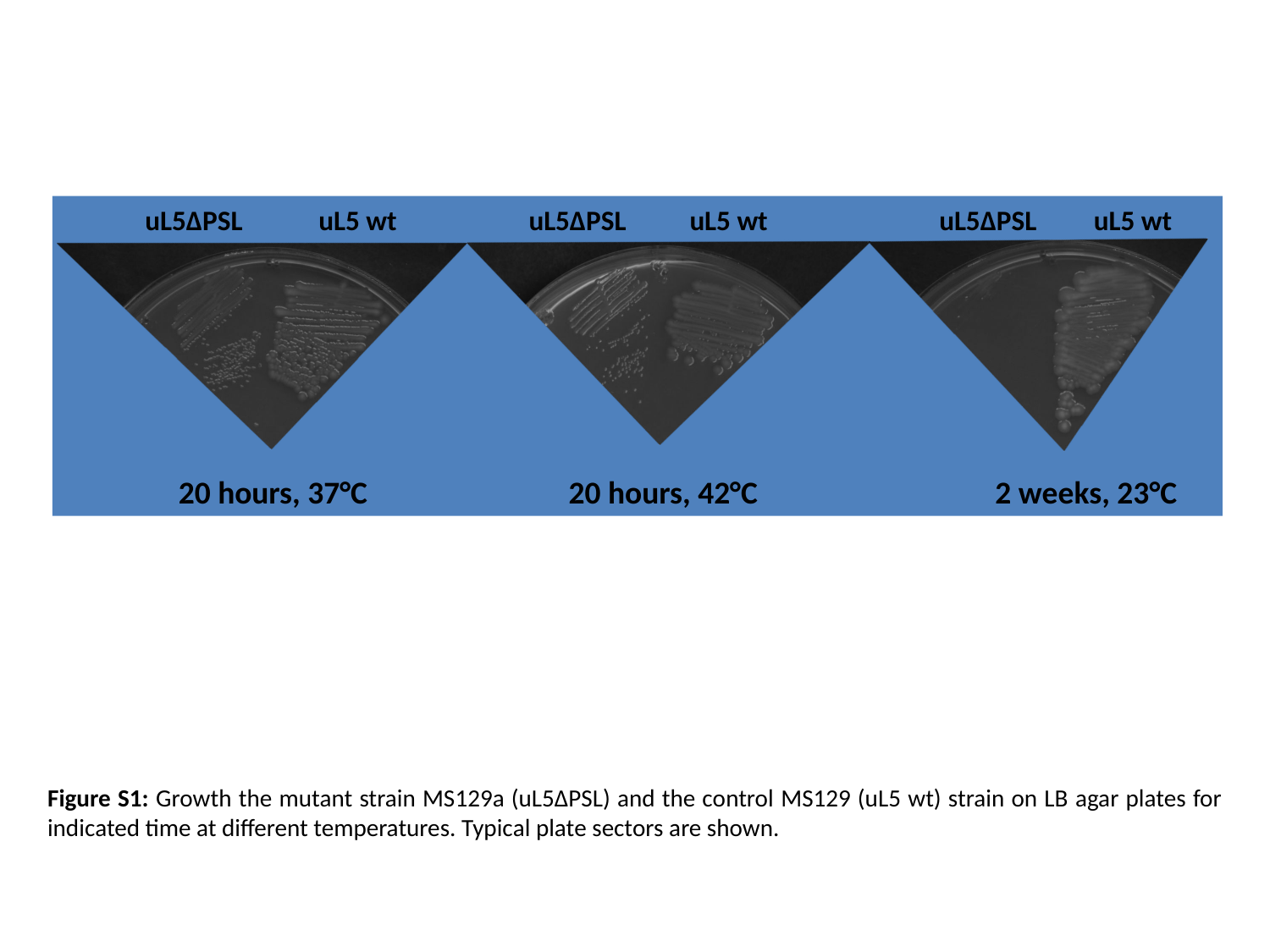

uL5ΔPSL uL5 wt
 uL5ΔPSL uL5 wt
 uL5ΔPSL uL5 wt
20 hours, 37°C
20 hours, 42°C
2 weeks, 23°C
Figure S1: Growth the mutant strain MS129a (uL5ΔPSL) and the control MS129 (uL5 wt) strain on LB agar plates for indicated time at different temperatures. Typical plate sectors are shown.

## Slide 2
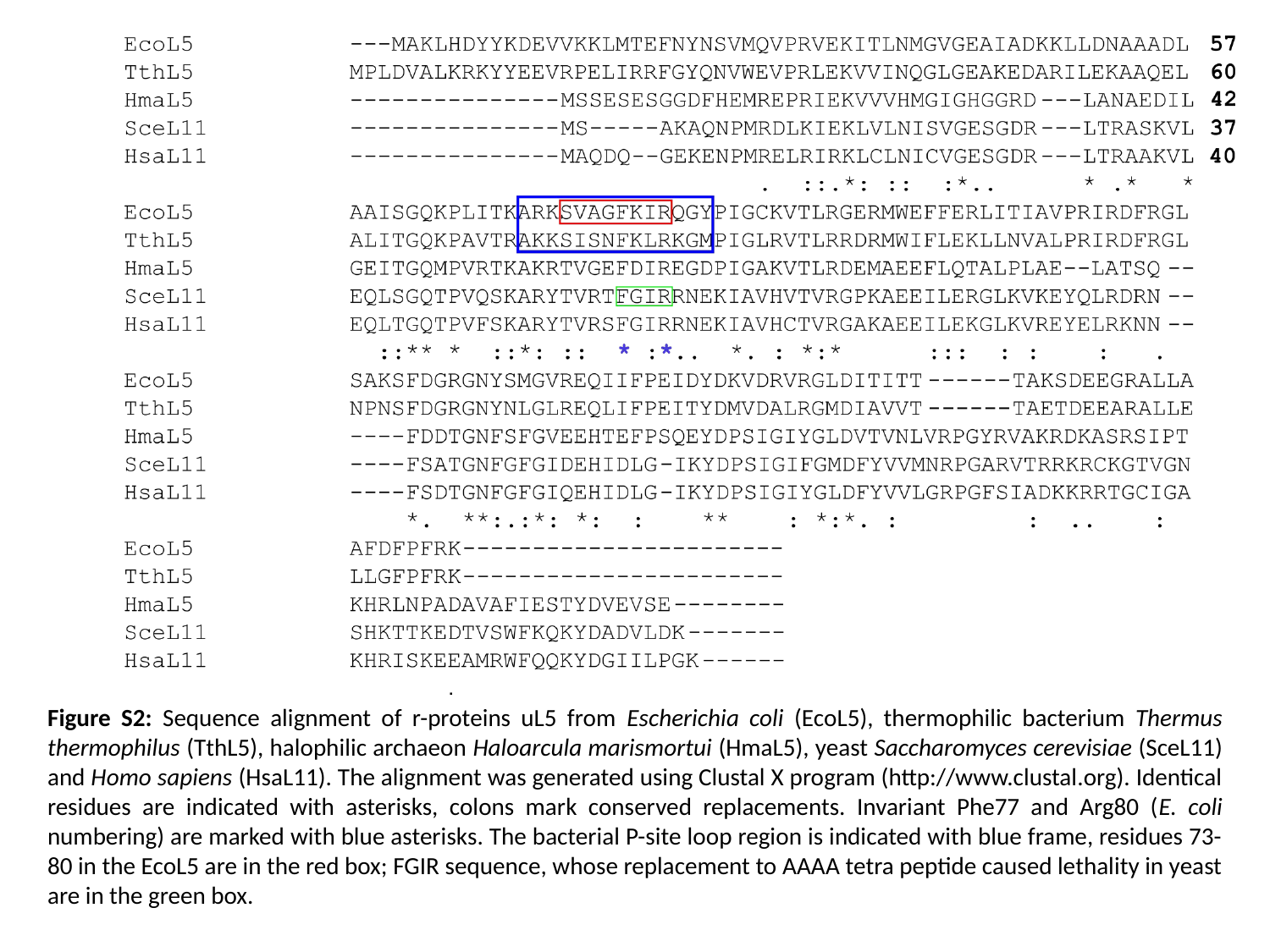

Figure S2: Sequence alignment of r-proteins uL5 from Escherichia coli (EcoL5), thermophilic bacterium Thermus thermophilus (TthL5), halophilic archaeon Haloarcula marismortui (HmaL5), yeast Saccharomyces cerevisiae (SceL11) and Homo sapiens (HsaL11). The alignment was generated using Clustal X program (http://www.clustal.org). Identical residues are indicated with asterisks, colons mark conserved replacements. Invariant Phe77 and Arg80 (E. coli numbering) are marked with blue asterisks. The bacterial P-site loop region is indicated with blue frame, residues 73-80 in the EcoL5 are in the red box; FGIR sequence, whose replacement to AAAA tetra peptide caused lethality in yeast are in the green box.

## Slide 3
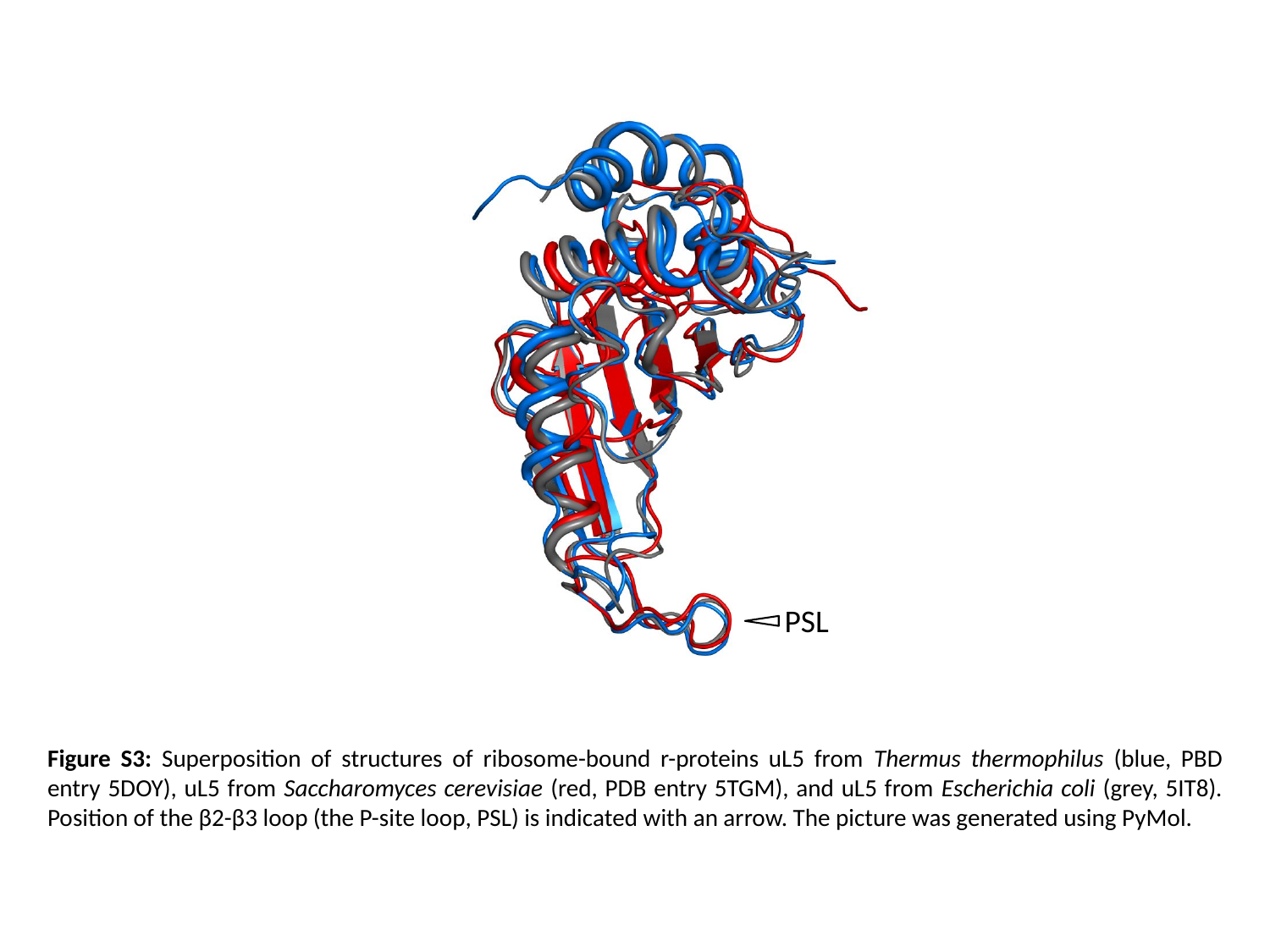

PSL
Figure S3: Superposition of structures of ribosome-bound r-proteins uL5 from Thermus thermophilus (blue, PBD entry 5DOY), uL5 from Saccharomyces cerevisiae (red, PDB entry 5TGM), and uL5 from Escherichia coli (grey, 5IT8). Position of the β2-β3 loop (the P-site loop, PSL) is indicated with an arrow. The picture was generated using PyMol.

## Slide 4
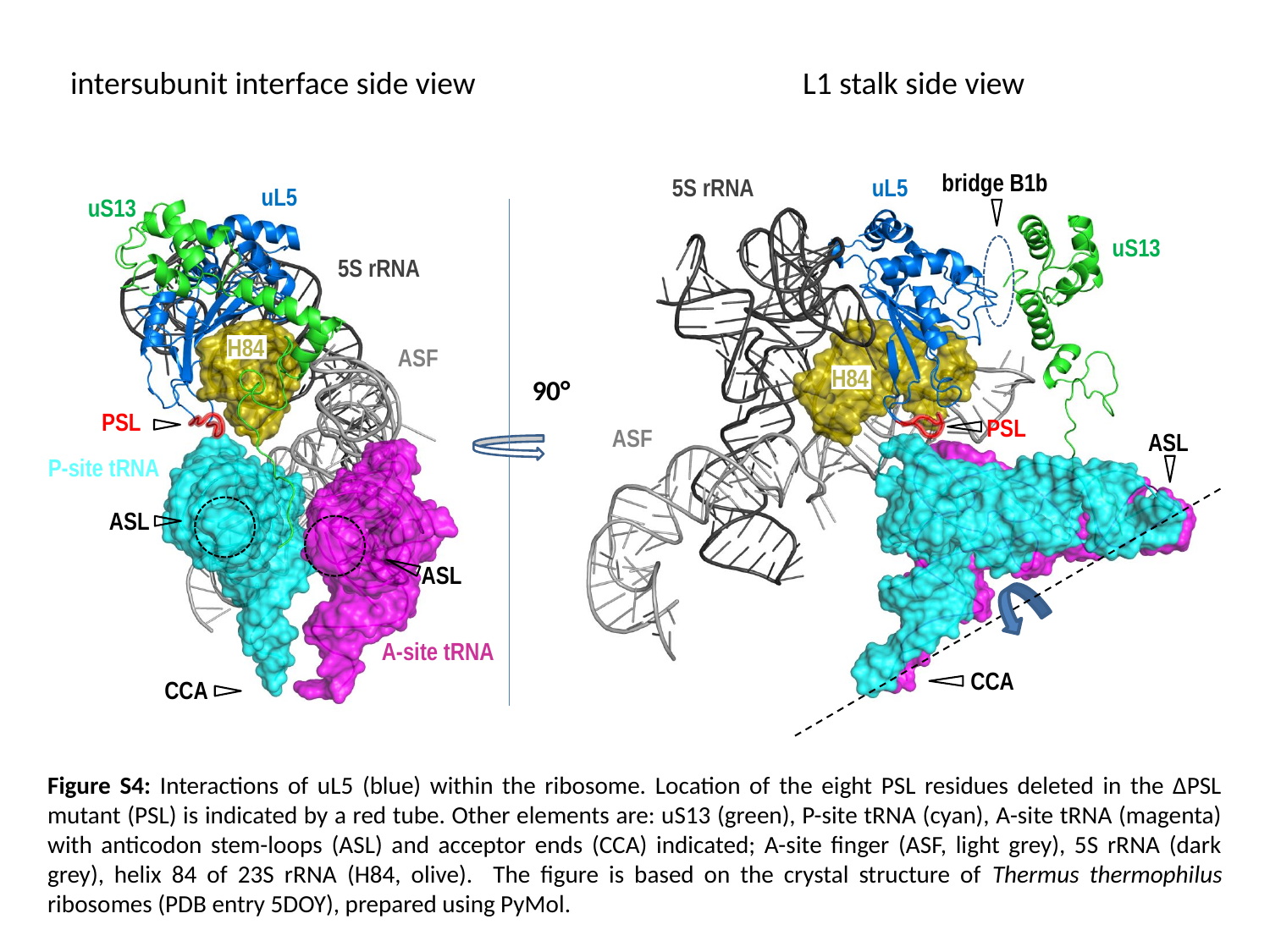

intersubunit interface side view
L1 stalk side view
bridge B1b
5S rRNA
uL5
uL5
uS13
uS13
5S rRNA
H84
ASF
H84
90°
PSL
PSL
ASF
ASL
P-site tRNA
ASL
ASL
A-site tRNA
CCA
CCA
Figure S4: Interactions of uL5 (blue) within the ribosome. Location of the eight PSL residues deleted in the ∆PSL mutant (PSL) is indicated by a red tube. Other elements are: uS13 (green), P-site tRNA (cyan), A-site tRNA (magenta) with anticodon stem-loops (ASL) and acceptor ends (CCA) indicated; A-site finger (ASF, light grey), 5S rRNA (dark grey), helix 84 of 23S rRNA (H84, olive). The figure is based on the crystal structure of Thermus thermophilus ribosomes (PDB entry 5DOY), prepared using PyMol.

## Slide 5
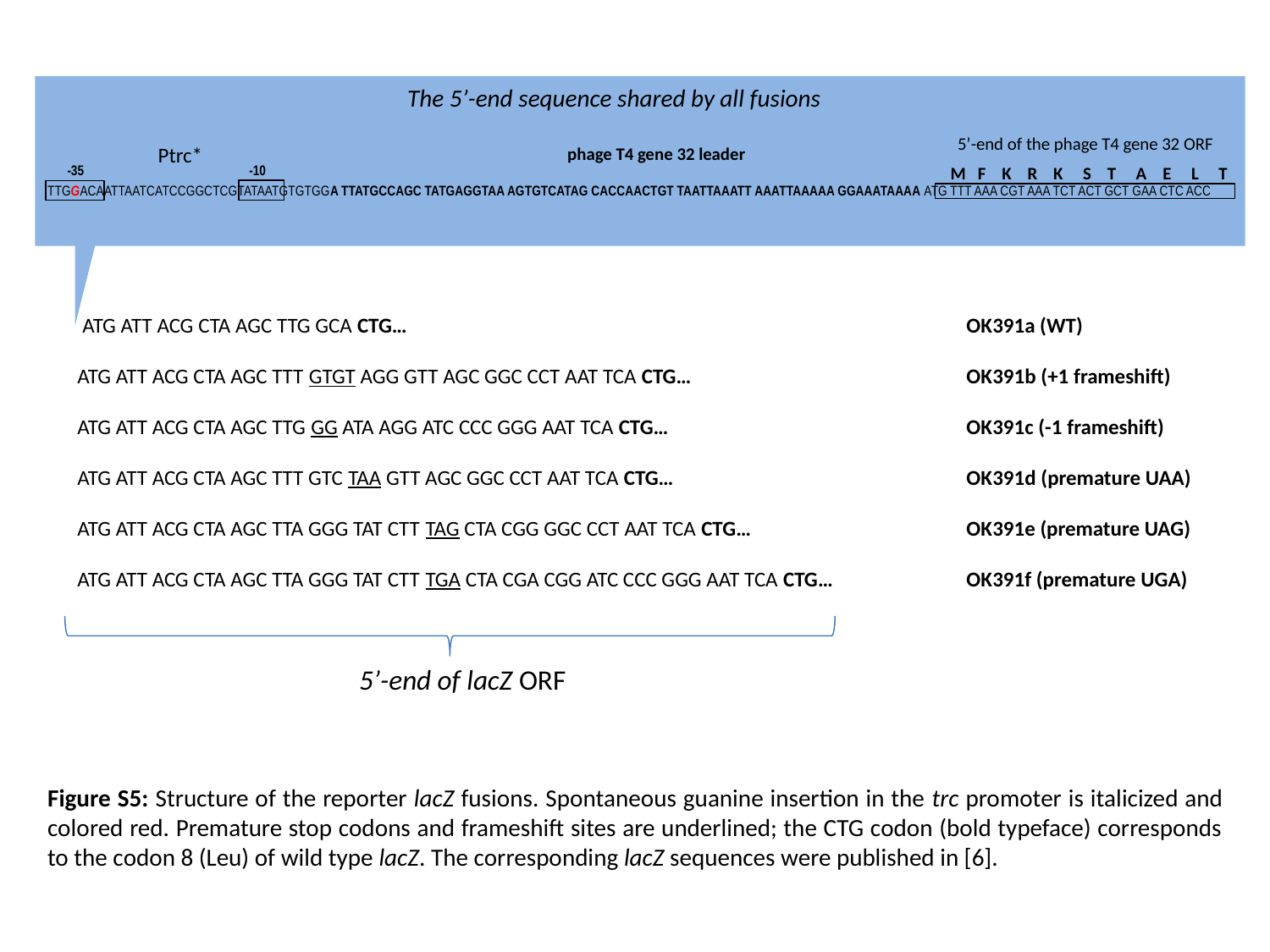

The 5’-end sequence shared by all fusions
5’-end of the phage T4 gene 32 ORF
Ptrc*
phage T4 gene 32 leader
-35 -10
M F K R K S T A E L T
TTGGACAATTAATCATCCGGCTCGTATAATGTGTGGA TTATGCCAGC TATGAGGTAA AGTGTCATAG CACCAACTGT TAATTAAATT AAATTAAAAA GGAAATAAAA ATG TTT AAA CGT AAA TCT ACT GCT GAA CTC ACC
 ATG ATT ACG CTA AGC TTG GCA CTG…					OK391a (WT)
ATG ATT ACG CTA AGC TTT GTGT AGG GTT AGC GGC CCT AAT TCA CTG…			OK391b (+1 frameshift)
ATG ATT ACG CTA AGC TTG GG ATA AGG ATC CCC GGG AAT TCA CTG… 		OK391c (-1 frameshift)
ATG ATT ACG CTA AGC TTT GTC TAA GTT AGC GGC CCT AAT TCA CTG…			OK391d (premature UAA)
ATG ATT ACG CTA AGC TTA GGG TAT CTT TAG CTA CGG GGC CCT AAT TCA CTG…		OK391e (premature UAG)
ATG ATT ACG CTA AGC TTA GGG TAT CTT TGA CTA CGA CGG ATC CCC GGG AAT TCA CTG…		OK391f (premature UGA)
5’-end of lacZ ORF
Figure S5: Structure of the reporter lacZ fusions. Spontaneous guanine insertion in the trc promoter is italicized and colored red. Premature stop codons and frameshift sites are underlined; the CTG codon (bold typeface) corresponds to the codon 8 (Leu) of wild type lacZ. The corresponding lacZ sequences were published in [6].
