# Supplementary material for: The P-Site Loop of the Universally Conserved Bacterial Ribosomal Protein L5 Is Required for Maintaining Both Translation Rate and Fidelity
Source: Int J Mol Sci. 2023 Sep 19;24(18):14285. doi: 10.3390/ijms241814285 (PMC10531944; doi:10.3390/ijms241814285)
Supplement: Supplementary file 1 [file ijms-24-14285-s001.zip › Supplementary tables.pdf]

Table S1: Translation fidelity in the control and ΔPSL strains

uL5 variant	*lacZ* variant	LacZ activity, Miller units	Standard deviation
wild type	WT	13943	±455
wild type	+1 frameshift	16.2	±1.8
wild type	-1 frameshift	3.0	±0.2
wild type	Premature UAA	0.5	±0.0
wild type	Premature UAG	6.9	±0.5
wild type	Premature UGA	8.9	±0.3
ΔPSL	WT	18703	±2303
ΔPSL	+1 frameshift	19.4	±0.6
ΔPSL	-1 frameshift	8.4	±0.5
ΔPSL	Premature UAA	2.1	±0.1
ΔPSL	Premature UAG	63.4	±10.2
ΔPSL	Premature UGA	46.8	±1.9

Table S2: Oligonucleotides used in the work

**Name**	**Usage**	**Sequence**	**Source**
AK07	The *lac* fusion check/sequencing	5’- GTGAACGCTCTCCTGAGTAGG	[30]
AK08b	The *lac* fusion check/sequencing	5’- CATTCGCCATTCAGGCTGCGCAAC	[30]
AK45	Constructing *lac* fusions	5’-GACGCCCGCCATAAACTGCCAGGAATTAAAATGAGACGTTCTGAAATGAGCTGTTGACAATTAATCATCC	This work
AK46	Constructing *lac* fusions	5’-GACACTTTACCTCATAGCTGGCATAATCCACACATTATACGAGCCGGATGATTAATTGTCAACAGC	This work
AK47	Constructing *lac* fusions	5’- AGTCACGACGTTGTAAAACGACGGCCAGTGAATCCGTAATAGCCATTTGTGCAGCGAGTTC	This work
AK226	Constructing *lac* fusions	5’-GCTTAGCGTAATCATGGTGAGTTCAGCAGTAGATTTACG	This work
L5delta73-80F	73-80 deletion	5’-CACCAAAGCACGCAAACAGGGCTATCCGATC	This work
L5delta73-80R	73-80 deletion	5’-GATCGGATAGCCCTGTTTGCGTGCTTTGGTG	This work
rplE34cat-F1	*rplE* knockout, forward	5’-CTACAATTCTGTCATGCAAGTCCCTCGGGTCGAGAAGATCATGGAGAAAAAAATCACTGGAT	This work
rplEcat-R1	*rplE* knockout, reverse	5’-CTTTCATTGATTGCTTAGCCATTTAGTAACCCTACCTTACGCCCCGCCCTGCCACTC	This work
rplE-check-F	knockout verification, sequencing	5’-TGTAGGCTTTAGATTCGAAGACGG	[2]
rplE-check-R	knockout verification, sequencing	5’-CGCTTTCAGTTCAGCGCGTTTCGC	[2]
rplE_rec_F	Restoring chromosomal *rplE*	5’-ATGGCGAAACTGCATGAT	This work
rplE_rec_R	Restoring chromosomal *rplE*	5’-CGGTGGTATATCCAGTGATTTTTTTCTCCATGATCTTCTCCACTTACTTGCGGAACGGGAAG	This work

Table S3: *Escherichia coli* strains and plasmids used in the work

Name	Genotype/description	Reference/source
**Strains**		
OK391a	MS02 *lacI'::bla-Ptrc*-g32-lacZ-1* (wt)	this work
OK391b	MS02 *lacI'::bla-Ptrc*-g32-lacZ-2* (+1 frameshift)	this work
OK391c	MS02 *lacI'::bla-Ptrc*-g32-lacZ-3* (-1 frameshift)	this work
OK391d	MS02 *lacI'::bla-Ptrc*-g32-lacZ-4* (premature UAA)	this work
OK391e	MS02 *lacI'::bla-Ptrc*-g32-lacZ-5* (premature UAG)	this work
OK391f	MS02 *lacI'::bla-Ptrc*-g32-lacZ-6* (premature UGA)	this work
OK392a	MG1655 *lacI'::bla-Ptrc*-g32-lacZ-1* (wt)	this work
OK392b	MG1655 *lacI'::bla-Ptrc*-g32-lacZ-2* (+1 frameshift)	this work
OK392c	MG1655 *lacI'::bla-Ptrc*-g32-lacZ-3* (-1 frameshift)	this work
OK392d	MG1655 *lacI'::bla-Ptrc*-g32-lacZ-4* (premature UAA)	this work
OK392e	MG1655 *lacI'::bla-Ptrc*-g32-lacZ-5* (premature UAG)	this work
OK392f	MG1655 *lacI'::bla-Ptrc*-g32-lacZ-6* (premature UGA)	this work
OK404a	OK392**a** *rplE1::cat* (wild type uL5)	this work
OK404b	OK392**b** *rplE1::cat* (wild type uL5)	this work
OK404c	OK392**c** *rplE1::cat* (wild type uL5)	this work
OK404d	OK392**d** *rplE1::cat* (wild type uL5)	this work
OK404e	OK392**e** *rplE1::cat* (wild type uL5)	this work
OK404f	OK392**f** *rplE1::cat* (wild type uL5)	this work
OK406a	OK392**a** *rplEΔ73-80::cat* (uL5ΔPSL)	this work
OK406b	OK392**b** *rplEΔ73-80::cat* (uL5ΔPSL)	this work
OK406c	OK392**c** *rplEΔ73-80::cat* (uL5ΔPSL)	this work
OK406d	OK392**d** *rplEΔ73-80::cat* (uL5ΔPSL)	this work
OK406e	OK392**e** *rplEΔ73-80::cat* (uL5ΔPSL)	this work
OK406f	OK392**f** *rplEΔ73-80::cat* (uL5ΔPSL)	this work
MG1655	*rph-1*	wild type *E. coli* [28]
MS02	*gal490 ldcI857 Δ(cro-bioA) lacI'::bla-cat-sacB-lacZYA*	[30]
MS45	MS02 *lacI'::bla-Ptrc-g32-lacZYA*	this work
MS45a	MS02 *lacI'::bla-Ptrc*-g32-lacZYA* (G insertion into the -35 promoter sequence)	this work
MS128/pNK12	NM300 *ΔrplE34::cat* (transformed with the complementing plasmid pNK12)	this work
MS129	MG1655 *rplE1::cat* (wild type uL5)	this work

MS129a	MG1655 *rplEΔ73-80::cat* (uL5ΔPSL)	this work
NM300	MG1655 *lacX74 mini-λ-Tet*	N. Majdalani (NIH)
**Plasmids**		
pACYC184	cloning vector, Tet^R, Cam^R	NEB, [26]
pNK12	arabinose-induced expression vector with cloned wild type *rplE* ORF, Amp^R	[3]
pNK14	same as pNK12 with mutated *rplE* ORF encoding uL5ΔPSL	this work
pSG25	*lacZ* (wild type)	[7]
pSGlac7	*lacZ* (+1 frameshift)	[7]
pSG12DP	*lacZ* (-1 frameshift)	[7]
pSG853	*lacZ* (premature UAA stop codon)	[7]
pSG163	*lacZ* (premature UAG stop codon)	[7]
pSG3/4	*lacZ* (premature UGA stop codon)	[7]
